# Towards Addressing the Body Electrolyte Environment via Sweat Analysis:Pilocarpine Iontophoresis Supports Assessment of Plasma Potassium Concentration

**DOI:** 10.1038/s41598-017-12211-y

**Published:** 2017-09-18

**Authors:** Donato Vairo, Laurie Bruzzese, Marion Marlinge, Lea Fuster, Nabil Adjriou, Nathalie Kipson, Philippe Brunet, Jennifer Cautela, Yves Jammes, Giovanna Mottola, Stephane Burtey, Jean Ruf, Regis Guieu, Emmanuel Fenouillet

**Affiliations:** 1 UMR MD2, Aix Marseille University, Marseille, France; 2 Laboratory of Biochemistry, Timone Hospital, Marseille, France; 3 Department of Dialysis, Conception Hospital, Marseille, France; 4 INSERM, U 1076 Marseille, France; 5 Department of Cardiology, Nord Hospital, Marseille, France; 6 INSERM, Paris, France; 7 CNRS, Institut des Sciences Biologiques, Paris, France

## Abstract

Electrolyte concentration in sweat depends on environmental context and physical condition but also on the pathophysiological status. Sweat analyzers may be therefore the future way for biological survey although how sweat electrolyte composition can reflect plasma composition remains unclear. We recruited 10 healthy subjects and 6 patients to have a broad range of plasma electrolyte concentrations (chloride, potassium and sodium) and pH. These variables were compared to those found in sweat produced following cycling exercise or pilocarpine iontophoresis, a condition compatible with operating a wearable device. We found no correlation between plasma and sweat parameters when exercise-induced sweat was analyzed, and we could identify a correlation only between plasma and sweat potassium concentration (R = 0.78, p < 0.01) when sweat was induced using pilocarpine iontophoresis. We tested measurement repeatability in sweat at 24hr-interval for 3 days in 4 subjects and found a great intra-individual variability regarding all parameters in exercise-induced sweat whereas similar electrolyte levels were measured in pilocarpine-induced sweat. Thus, electrolyte concentration in sweat sampled following physical activity does not reflect concentration in plasma while pilocarpine iontophoresis appears to be promising to reproducibly address sweat electrolytes, and to make an indirect evaluation of plasma potassium concentration in chronic kidney disease and arrhythmia.

## Introduction

Sweat composition varies depending on environmental context, physical condition and body regulatory systems but also on the pathophysiological status^1–3^. Sweat analysis is considered as the future way for biological survey^1–5^: sweat can be collected *in vivo* via non-invasive means using easily wearable devices, and sweat is assumed to reflect the body’s biochemical environment. This would be of special interest when continuous monitoring is necessary, in ambulatory conditions or for out-door uses. Sweat sample processing is indeed generally simple and does not require any treatment prior to analysis. Yet, a main limitation of the use of sweat in a context of *in vivo* monitoring is production of a sufficient volume of sweat for analysis^1,2,6,7^.

Sweat analysis is already routinely used for the diagnosis of cystic fibrosis^8–10^ and testing drug or ethanol consumption^2,11^. Sweat analysis is also currently developed to monitor diabetic, schizophrenic and tumorigenic prognostic markers^2^. Many efforts are therefore ongoing to design fast, reliable and user-friendly methods to access the body’s biochemical status via sweat, and hence to directly address pathophysiological conditions. One such attempt is development of alternative approaches of non-invasive collection and manipulation of metabolites released in sweat by skin via the use of osmotic properties of hydrogels contained in micropatches^12–14^. These approaches are based on nanosized hydrogel particles functionalized with affinity reactive baits and are designed to investigate complex body fluids such as sweat. Those formats can concentrate up to 10^4^-fold and isolate in one single step low abundance biomarkers prior to analysis whereas unwanted high abundance analytes are excluded concomitantly^12–14^.

A major progress in the development of a monitoring equipment was recently made. A group described an innovative colorimetric approach to measure various biological parameters (pH, sodium, lactates and glucose) in sweat samples collected in a context of physical exercise performed to increase sweat sample volume^5^. In this work, the authors obtained comparable results using the sweat-based colorimetric procedure they described and standard biochemical techniques used to analyse sweat. Their report, the last of several others in the field (reviewed in ref.^1,3^), is quite significant and promising as it describes a device providing embedded chemical analyses. The article raises however at least two important issues.

First, inducing sweat production is an essential step in the development of sweat-based monitoring devices^1,2,6,7^ but the influence of this step on sweat composition is rarely considered. Sweat sample volume is generally increased using physical exercise. Exercise modifies however sweat composition^1,15,16^ and may interfere with the use of sweat to address steady-state physiological parameters and their pathological variations. Sweat production is also sometimes induced using application on skin of pharmacological compounds such as pilocarpine^17^. This alkaloid agent stimulates sweat gland secretion and is routinely used via iontophoresis in cystic fibrosis diagnosis for sweat chloride titration^8,17^. There are obviously many reasons why pilocarpine iontophoresis may influence sweat characteristics^1^. Additionally, each of these two common procedures probably modifies sweat composition in a different manner.

Second, the literature in the field including its latest reports^3–5^ often takes for granted that sweat composition reflects plasma composition but such relationship remains unclear. Yet, following storage in the sweat glands, secretion into the sweat and transport to the skin surface, sodium and chloride are for example partially and selectively reabsorbed during transportation. These mechanisms can be differently affected by various disorders^2,18,19^.

In this study, we addressed two key-points in the development of a reliable sweat-based monitoring of the body’s biochemical composition: i) how physical exercise and pilocarpine iontophoresis influence electrolyte concentration and pH of sweat? ii) can sweat analysis address plasma electrolyte levels?

## Results

### Subject panel

Ten healthy subjects and 6 patients were selected according to their plasma electrolyte levels to constitute a panel encompassing a broad range of values (see Table 1, “Pilocarpine” condition, column “Plasma: Mean ± SD, [range]”), and hence to take account levels routinely found in clinical settings. We analyzed sweat sample volumes that were ≈90 µL.

**Table 1:** Electrolyte concentrations and pH values in plasma and sweat of subjects: analysis. Sweat production was induced by cycling exercice in healthy subjects only (n = 10), and by pilocarpine iontophoresis in both healthy subjects and patients with end stage kidney disease (n = 6). Variables are expressed as mean ± standard deviation (SD) and range as indicated. Correlations were analysed using Pearson rank correlation coefficient. Linear regression was assessed and r2 value determined. A p value < 0.05 was considered significant.

	Exercice (n = 10)					Pilocarpine (n = 16)				
	Plasma Mean ± SD [range]	Sweat Mean ± SD [range]	Pearson’s r	R2	P value	Plasma Mean ± SD [range]	Sweat Mean ± SD [range]	Pearsons’s r	R2	P value
Chloride (mM)	101 ± 2 98–105]	45 ± 7 31–56]	−0.21	0.04	0.56	99 ± 4 [90–105]	40 ± 8 [26–51]	−0.32	0.10	0.21
Potassium (mM)	4.3 ± 0.5 [3.7–5.0]	5.4 ± 2.1 2.7–9.0]	0.13	0.02	0.7	4.7 ± 0.9 [3.4–6.9]	8.9 ± 1.2 7.0–11.2]	0.78	0.61	0.0003
Sodium (mM)	139 ± 3 [134–146]	36 ± 13 [11–51]	0.07	0.005	0.84	134 ± 9 [118–145]	50 ± 20 [25–80]	−0.069	0.005	0.80
pH	7.38 ± 0.03 [7.35–7.43]	7.27 ± 0.31 [6.90–7.70]	0.08	0.006	0.82	7.38 ± 0.03 [7.35–7.45]	7.33 ± 0.30 [6.9–7.79]	−0.40	0.16	0.12

### Sweat induced by physical exercise

**Figure 1:**
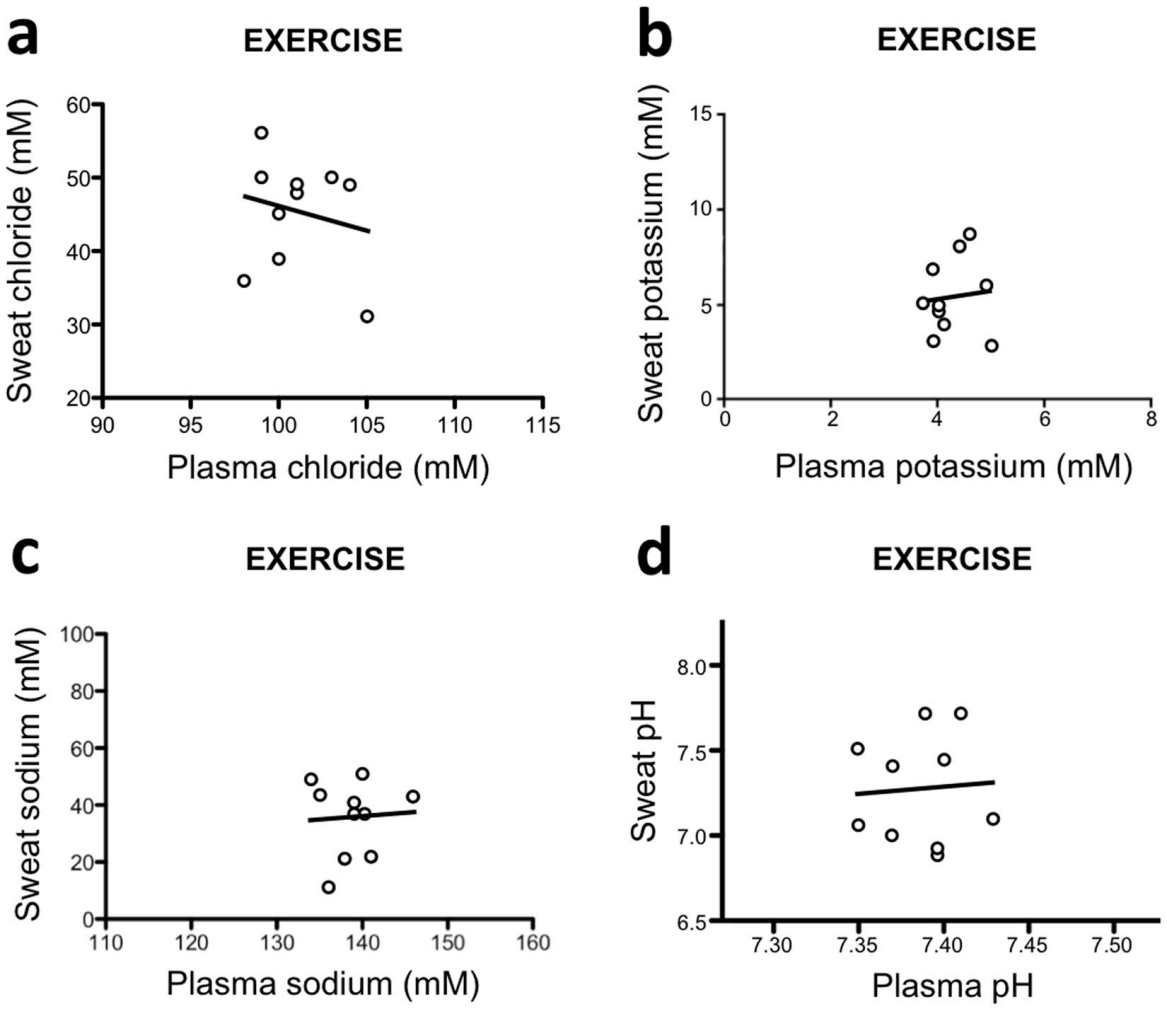
Relationship between electrolyte levels or pH in blood and sweat produced following physical exercise. Healthy subjects performed cycling exercise (120 W; 20 min). Blood samples (3 mL) were obtained via venous puncture. Sweat samples (90 microL) were collected in the lower back using a FinnPipette^TM^. Levels of chloride (**a**), potassium (**b**) and sodium (**c**) as well as pH (**d**) were measured using a Cobas^TM^ 8000 apparatus.

First, we compared plasma and sweat levels of electrolytes following physical exercise (Fig. 1), which was performed for obvious reasons in healthy subjects only. No correlation was found between plasma and sweat level of chloride, potassium or sodium as well as between plasma and sweat pH values (Table 1).

### Sweat induced by pilocarpine iontophoresis

**Figure 2:**
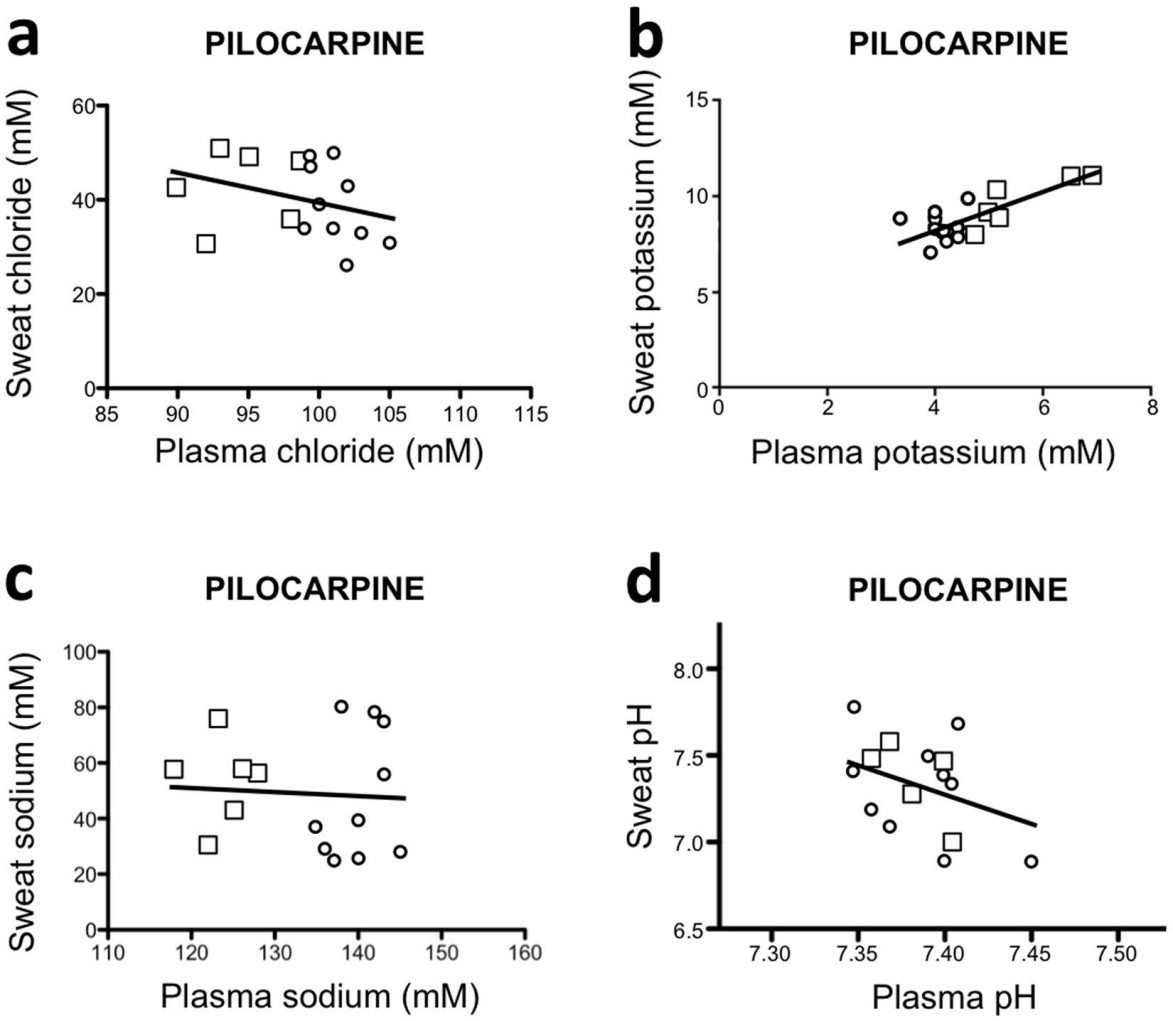
Relationship between electrolyte levels or pH in blood and sweat produced following pilocarpine iontophoresis. Sweat production was obtained using pilocarpine iontophoresis via the Macroduct^TM^ system. Blood samples (3 mL) were obtained following venous puncture. Sweat samples (90 microL) were collected (healthy = small circle; patient = small square). Levels of chloride (**a**), potassium (**b**) and sodium (**c**) as well as pH (**d**) were measured using a Cobas^TM^ 8000 apparatus.

Following pilocarpine iontophoresis in healthy subjects and patients (Fig. 2), a correlation was found only between plasma and sweat level of potassium. No correlation was found regarding plasma and sweat level of chloride and sodium as well as between plasma and sweat pH values (Table 1).

### Measurement repeatability

**Figure 3:**
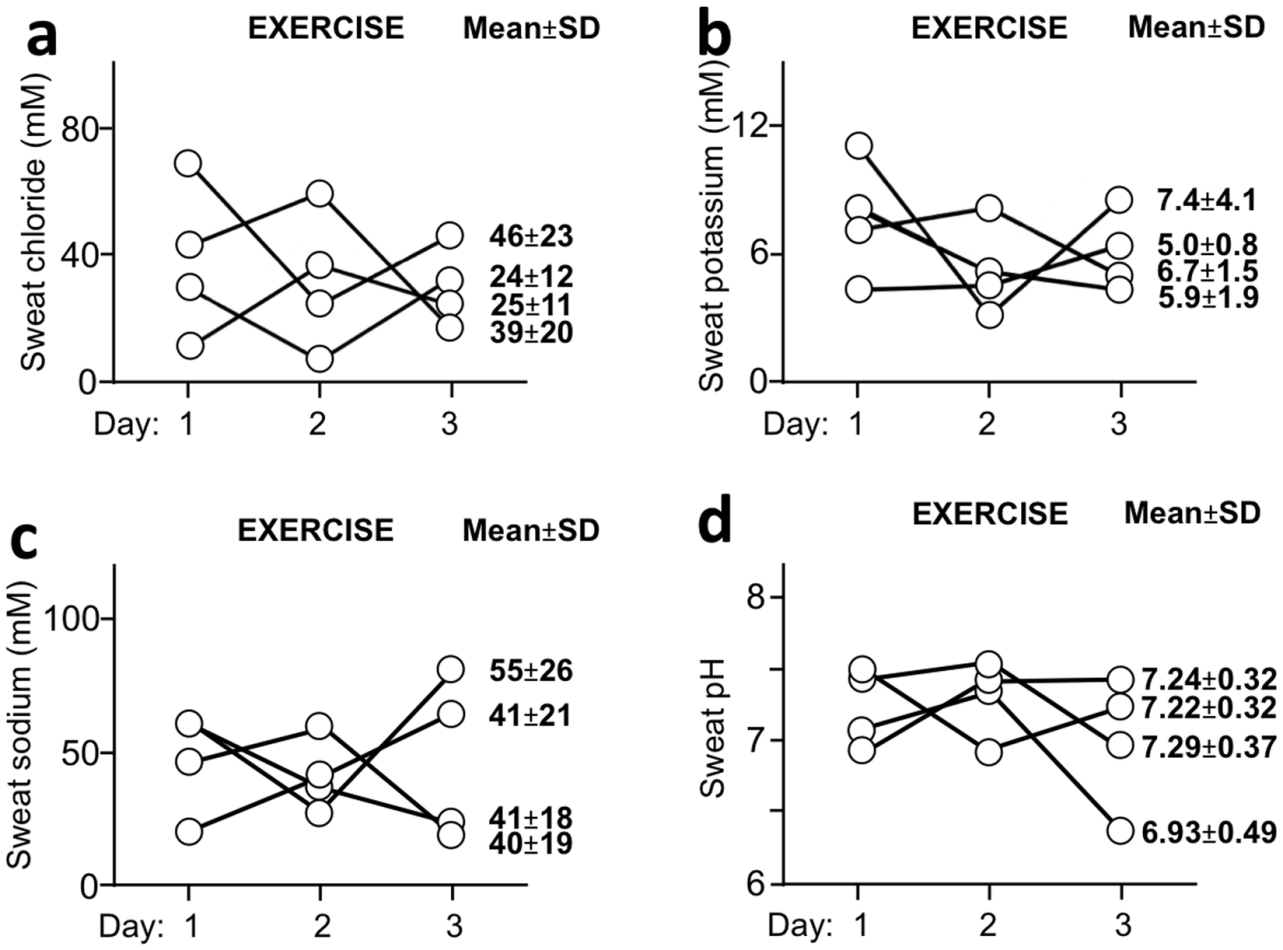
Measurement repeatability of various parameters in sweat produced following cycling exercise. We investigated the measurement repeatability of various parameters by measuring electrolyte levels or pH in 4 healthy subjects for 3 days at 24hr-interval (level of chloride: (**a**) potassium: (**b**) sodium: (**c**) pH: (**d**)). Mean ± SD data corresponding to the values obtained for 3 days for a given subject are indicated.

**Figure 4:**
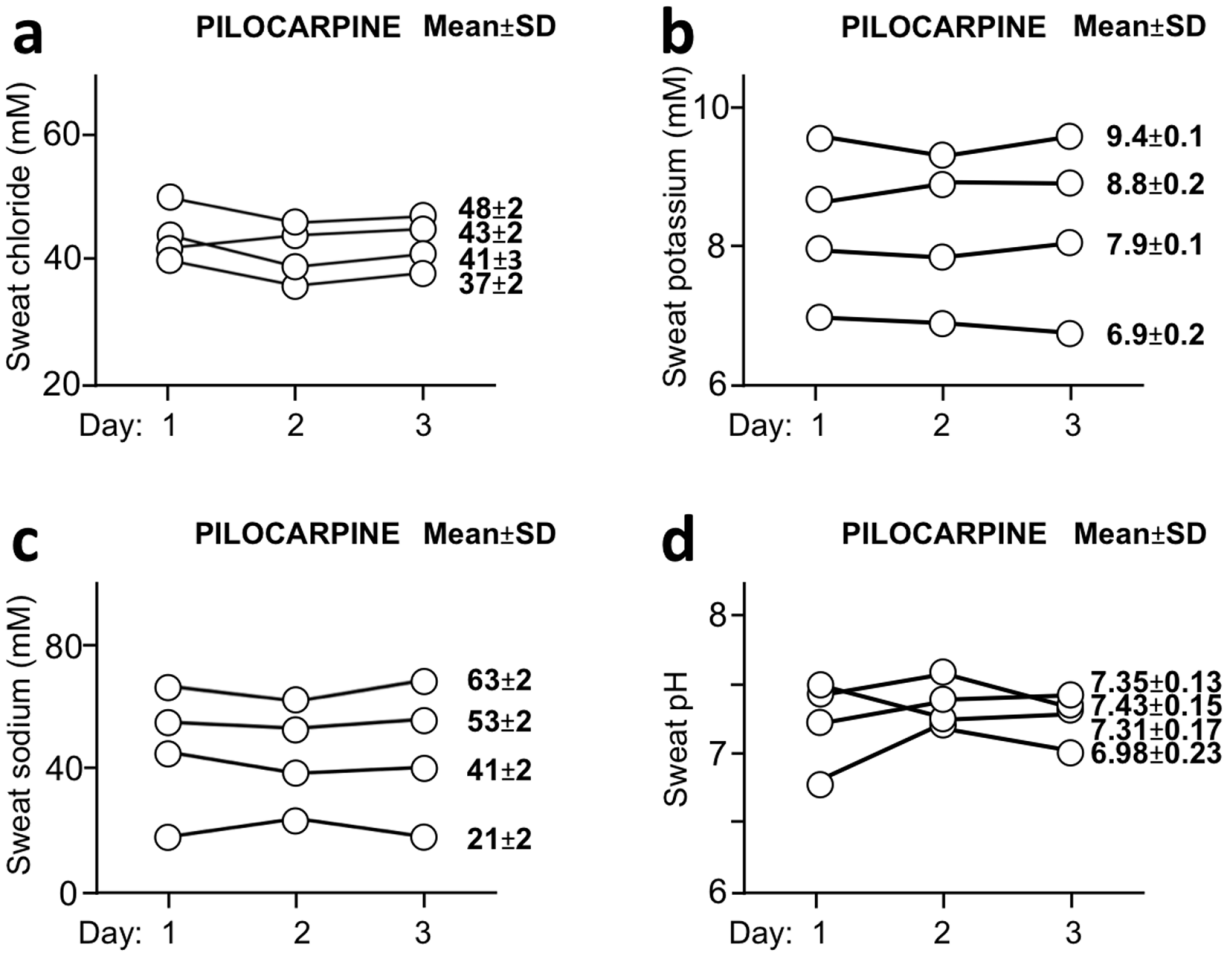
Measurement repeatability of various parameters in sweat produced following pilocarpine iontophoresis. We investigated the measurement repeatability of various parameters by measuring electrolyte levels or pH in 4 healthy subjects for 3 days at 24hr-interval (level of chloride: (**a**), potassium: (**b**), sodium: (**c**); pH: (**d**)). Mean ± SD data corresponding to the values obtained for 3 days for a given subject are indicated.

We investigated in 4 subjects the measurement repeatability of the various parameters in sweat by measuring the variables at 24 hours interval for 3 days. When physical exercise was used to induce sweat (Fig. 3), a great intra-individual variability was found for all parameters. When pilocarpine stimulation was used (Fig. 4), measurements of concentration of chloride, potassium and sodium gave reproducible values whereas intra-individual variability was found regarding pH measurement.

## Discussion

We obtained here three main findings: (i) when pilocarpine iontophoresis was used to stimulate sweat production according to an established procedure followed for cystic fibrosis diagnosis, a correlation was found between plasma potassium level and sweat potassium level, no relationship being found regarding sweat and plasma levels for the other electrolytes and pH values; (ii) when cycling exercise was used to stimulate sweat production, no correlation was found between sweat and plasma parameters; (iii) similar chloride, potassium and sodium concentrations were measured in sweat at 24hr-interval for 3 days using pilocarpine iontophoresis, which indicates measurement repeatability, whereas a great intra-individual variability was found following cycling exercise.

Sweat induced by pilocarpine iontophoresis results from local peripheral cholinergic stimulation whereas central, peripheral and thermal mechanisms influence sweat rate and composition in a context of physical exercise^1^. Accordingly, the advantage gained by pilocarpine iontophoresis over physical exercise may be due to the fact that the alkaloid tends to unify sweating conditions among the subjects whereas native sweating during physical exercise is highly dependent on various individual parameters, including physical condition, body temperature, food, emotional state and environment^1,2,15,16^. These latter factors may be also responsible for the poor measurement repeatability found in our work using exercise-induced sweat whereas pilocarpine iontophoresis significantly improved repeatability, an important property for *in vivo* assays used in clinical settings.

The discrepancy observed here between plasma and sweat concentrations of most electrolytes but potassium is probably largely due to selective filtering of electrolytes inherent to the function of the sweat gland^1,2,18^, even following pilocarpine iontophoresis. Additional substantial reasons can be probably found in the presence within the skin of extrarenal mechanisms that regulate electrolyte homeostasis and act via sodium and chloride sequestration in the skin interstitium^20,21^. Finally, it is of note that the low inter-individual variability we found here further supports a previous study that reported low variability within subjects regarding sweat sodium level contrary to potassium level^7^.

Although the results we present here further highlight pilocarpine as a key component to produce sweat as part of ambulatory monitoring, it remains that the discrepancies found here between plasma and sweat levels of several electrolytes challenge the rapid marketing of sweat analyzers. Additional concerns include for example the fact that dehydration reflects a loss of water leading to hypertonicity^22^, which can be evaluated probably only by plasma osmolality or at least natremia^19^. In this case, simply considering increased sodium level in sweat as a marker for dehydration during a long run may lead to misinterpretation. Yet, a smartphone application was recently proposed to indirectly monitor hydration via pH changes in sweat and indicate during physical exercise the proper time for hydration^23^. Our results do not however support this use as we found here that sweat pH-measurements were not reproducible in the same subject in spite of similar exercise conditions.

Of particular interest is our finding that sweat potassium level was correlated with plasma potassium level. This point is quite interesting because reliable, non invasive monitoring of potassium concentration in the body is of paramount importance in a context of ambulatory monitoring as small changes in potassium affect heart rhythm level, for example in end-stage kidney disease patients^24,25^. Potassium concentration in sweat is often found, or considered, to be similar to plasma and poorly vary with sweat rate^1^. For some experts in the field, potassium level in final sweat > 8 mM may be therefore indicative of sample evaporation whereas for others usual potassium level in final sweat is in the 2–8/10 mM range (review^1^). We found here a rather high mean of sweat potassium levels, in agreement with others^7^ (≈13 mM) who additionally reported high inter-individual variability.

There are at least four limitations to our work: (i) we addressed a low number of subjects. Yet, if a routine sweat testing is expected to monitor plasma electrolyte level, then the data obtained should be consistent and relevant in the vast majority of subjects. Major discrepancies were manifest here although our panel was small. Thus we consider that our findings did not validate the proof of concept tested here except for plasma potassium level; (ii) sweat collected using occlusive devices may contain non native sweat electrolyte concentrations because of electrolyte leaching from the stratum corneum of the skin in these conditions^26^; (iii) we did not normalize sweat volume because we considered that the Macroduct system strongly limits per se evaporation; (iv) we did not take into account genders although it was found in particular that sodium level is lower in male sweat^6,7^.

Overall, the merit of this study lies in the fact that it addressed a generally neglected question by evaluating the relationship between plasma and sweat composition, this link being generally taken for granted. The results we present here indicate that electrolyte concentration in sweat sampled following physical activity does not properly reflect plasma electrolyte levels, and hence cannot be used without careful consideration for biological survey, in particular for monitoring hydration state. We show however that pilocarpine iontophoresis is a promising way to reproducibly address sweat electrolytes, and to make an indirect evaluation of plasma potassium level, a marker that is particularly relevant in arrhythmia and chronic kidney disease^24,25^.

## Subjects and Methods

We recruited 10 healthy subjects (6 men and 4 women; mean age 54 years; range 38–67) and 6 patients (4 men and 2 women; mean age 59 years; range 49–78; plasma potassium level: 5.7 ± 0.8 mM; plasma sodium level: 124 ± 4 mM) who were attending the hemodialysis center of the hospital to treat end-stage kidney disease (mean creatininemia: 220 ± 69 microM; cause of kidney disease: polycystic kidney disease: 1 patient, hypertension: 1 patient, diabetes mellitus: 4 patients), a disorder that is generally associated with abnormal plasma levels of electrolytes^19,24,25^. The protocol was approved by the Ethics Committee of our institution (CPP Sud Mediterranée) and the study was carried out in accordance with the Code of Ethics of the World Medical Association (Declaration of Helsinki). Procedures were carried out with the adequate understanding and written consent of the subjects.

### Induction of sweat production

Sweating was induced following physical exercise consisting in cycling exercise (for ethical reasons, exercise was performed by healthy subjects only). Prior to exercise, a medical interview was carried out to verify that all subjects had breakfast 3–4 hours before the cycling exercise and no special diets, pathological disorders or medication. After a resting period of 1 hour in an air conditioned room around 25 °C, cycling exercise was performed at this temperature between 10 and 11 h AM in conditions (120 w, 20 min) that enable sufficient sweating for sampling as determined as part of our practice of exercise stress test in cardiology settings. We collected sweat in the lower back region as often done^5,7^, using a FinnPipette^TM^ because we considered that this approach largely respects native sweating conditions. Alternatively, pilocarpine iontophoresis was performed in both healthy subjects and patients in the same time slot according to a standard procedure followed for cystic fibrosis diagnosis^17^. We made use of the Macroduct system 3700 (ELITechGroup) that delivers during 10 min a safe and optimal quantity of pilocarpine for gland stimulation (use of 0.5% pilocarpine nitrate; 1.5 mA). The skin was carefully cleaned prior to the experiment using soap and then tap water and a cotton pad prior to extensive rinsing with distilled water and drying with a sterile towel. Both electrodes were placed on the anterior size of the forearm as recommended by the manufacturer. Sweat collection was achieved using the Macroduct® sweat collection system, a device that consists of a concave disk and a spiral plastic tube that collects sweat. Whole blood samples (3 mL) were collected in parallel from the cubital vein at the end of the cycling period when cycling exercise was used to induce sweat production, or at the beginning of sweat production when pilocarpine stimulation was used.

### Measurement repeatability

To test reproducibility, 4 healthy subjects (3 men and 1 woman; mean age: 48 years; range: 38–65) performed the cycling exercise (see above) at 24 hours interval for 3 days and electrolytes were measured as described above. The same protocol was also followed by replacing the cycling exercise with pilocarpine stimulation.

### Biochemical analysis

Plasma and sweat electrolytes were measured using a Cobas 8000 analyzer (Roche Diagnostics®) and dedicated programs (range of linearity: chloride: plasma: 60–140 mM, sweat: 20–60 mM; potassium: plasma: 1.5–10 mM, sweat: 3–100 mM; sodium: plasma: 60–350 mM, sweat: 20–60 mM; intra-assay coefficient of variation: electrolytes: < 0.5% in sweat samples, < 1% in blood samples; pH: < 0.2% in all specimens). Specimen values higher than upper limit of the range were assessed following dilution.

### Statistical analysis

Quantitative variables were expressed as mean ± standard deviation (SD) and range as indicated. Correlations between biological parameters were quantified and tested using Pearson rank correlation coefficient. Linear regression was assessed and r2 value determined. A p value < 0.05 was considered significant. Analysis was performed using Prism, a GraphPad Software.

### Data availability statement

all data presented in this study are available upon request.we thank the AMIDEX foundation.

## References

[1] Baker LB (2017). Sweating rate and sweat sodium concentration in athletes: a review of methodology and intra/interindividual variability. Sports Med..

[2] Mena-Bravo A, de Castro L (2014). M. D. Sweat: a sample with limited present applications and promising future in metabolomics. J. Pharm. Biomed. Anal..

[3] Jadoon S (2015). Recent developments in sweat analysis and its applications. Int. J. Anal. Chem..

[4] Gao W (2016). Fully integrated wearable sensor arrays for multiplexed *in situ* perspiration analysis. Nature.

[5] Koh A (2016). A soft, wearable microfluidic device for the capture, storage, and colorimetric sensing of sweat. Sci. Transl. Med..

[6] Baker LB, Stofan JR, Hamilton AA, Horswill CA (2009). Comparison of regional patch collection vs. whole body washdown for measuring sweat sodium and potassium loss during exercise. J. Appl. Physiol..

[7] Appenzeller BM, Schummer C, Rodrigues SB, Wennig R (2007). Determination of the volume of sweat accumulated in a sweat-patch using sodium and potassium as internal reference. J. Chromatogr. B Analyt. Technol. Biomed. Life Sci..

[8] Saint-Criq VM, Gray A (2017). Role of CFTR in epithelial physiology. Cell. Mol. Life Sci..

[9] Cole DE, Boucher MJ (1986). Use of a new sample-collection device (Macroduct) in anion analysis of human sweat. Clin. Chem..

[10] Gonzalo-Ruiz J (2009). Early determination of cystic fibrosis by electrochemical chloride quantification in sweat. Biosens. Bioelectron..

[11] Selvam AP, Muthukumar S, Kamakoti V, Prasad S (2016). A wearable biochemical sensor for monitoring alcohol consumption lifestyle through Ethyl glucuronide (EtG) detection in human sweat. Sci. Rep..

[12] Magni R, Luchini A (2017). Application of hydrogel nanoparticles for the capture, concentration, and preservation of low-abundance biomarkers. Methods Mol. Biol..

[13] Shay T, Dickey MD, Velev OD (2017). Hydrogel-enabled osmotic pumping for microfluidics: towards wearable human-device interfaces. Lab. Chip..

[14] Dutkiewicz EP, Lin JD, Tseng TW, Wang YS, Urban PL (2014). Hydrogel micropatches for sampling and profiling skin metabolites. Anal. Chem..

[15] Fukumoto T (1988). Differences in composition of sweat induced by thermal exposure and by running exercise. Clin. Cardiol..

[16] Henkin SD, Sehl PL, Meyer F (2010). Sweat rate and electrolyte concentration in swimmers, runners, and nonathletes. Int. J. Sports Physiol. Perform..

[17] Coury J, Fogt EJ, Norenberg MS, Untereker DF (1983). Development of a screening system for cystic fibrosis. Clin. Chem.

[18] Cui CY, Schlessinger D (2015). Eccrine sweat gland development and sweat secretion. Exp. Dermatol..

[19] Sterns RH (2015). Disorder of plasma sodium –causes, consequences and corrections. N. Engl. J. Med..

[20] Machnik A (2010). Mononuclear phagocyte system depletion blocks interstitial tonicity-responsive enhancer binding protein/vascular endothelial growth factor C expression and induces salt-sensitive hypertension in rats. Hypertension..

[21] Wiig H (2013). Immune cells control skin lymphatic electrolyte homeostasis and blood pressure. J. Clin. Invest..

[22] Bhave G, Neilson EG (2011). Volume depletion versus dehydration: how understanding the difference can guide therapy. Am. J. Kidney Dis..

[23] Oncescu V, O’Dell D, Erickson D (2013). Smartphone based health accessory for colorimetric detection of biomarkers in sweat and saliva. Lab. Chip..

[24] Dunn JD, Benton WW, Orozco-Torrentera E, Adamson RT (2015). The burden of hyperkalemia in patients with cardiovascular and renal disease. Am. J. Manag. Care..

[25] Corsi C (2017). Noninvasive quantification of blood potassium concentration from ECG in hemodialysis patients. Sci. Rep..

[26] Weschler LB (2008). Sweat electrolyte concentrations obtained from within occlusive coverings are falsely high because sweat itself leaches skin electrolytes. J. Appl. Physiol..

